# Supplementary material for: Stacking Model‐Based Classifiers for Dealing With Multiple Sets of Noisy Labels
Source: Biom J. 2025 Mar 12;67(2):e70042. doi: 10.1002/bimj.70042 (PMC11898607; doi:10.1002/bimj.70042)
Supplement: Supplementary file 1 — Supporting Information [file BIMJ-67-e70042-s001.zip › density-based-ensemble-model-main/competitors_real_data/netlab-master/netlab-master/help/demgmm2.htm]

Netlab Reference Manual demgmm1


# demgmm1

## Purpose

Demonstrate density modelling with a Gaussian mixture model.

## Synopsis

```
demgmm1
```

## Description

The problem consists of modelling data generated by a mixture of three
Gaussians in 2 dimensions. The priors are 0.3,
0.5 and 0.2; the centres are (2, 3.5), (0, 0) and (0,2); the variances
are 0.2, 0.5 and 1.0. The first figure contains a
scatter plot of the data.

A Gaussian mixture model with three components is trained using EM. The
parameter vector is printed before training and after training. The user
should press any key to continue at these points. The parameter vector
consists of priors (the column), centres (given as (x, y) pairs as
the next two columns), and variances (the last column).

The second figure is a 3 dimensional view of the density function, while
the third shows the 1-standard deviation circles for the three components of
the mixture model.

## See Also

`gmm`, `gmminit`, `gmmem`, `gmmprob`, `gmmunpak`

---

**Pages:**
Index


---

Copyright (c) Ian T Nabney (1996-9)
